# Evolution of fragile X syndrome and neurodevelopmental disorders research: a Scopus-based bibliometric analysis

**DOI:** 10.12701/jyms.2026.43.44

**Published:** 2026-07-07

**Authors:** Wan Nur Amalina Zakaria, Nazihah Mohd Yunus, Aziati Azwari Annuar, Ahmad Aizat Abd. Aziz, Nurfadhlina Musa, Maruzairi Husain, Rowani Mohd Rawi

**Affiliations:** 1 Human Genome Centre, School of Medical Sciences, Health Campus, Hospital Pakar Universiti Sains Malaysia, Kota Bharu, Malaysia; 2 Department of Psychiatry, School of Medical Sciences, Health Campus, Hospital Pakar Universiti Sains Malaysia, Kota Bharu, Malaysia; 3 Department of Paediatrics, School of Medical Sciences, Health Campus, Hospital Pakar Universiti Sains Malaysia, Kota Bharu, Malaysia

**Keywords:** Autistic disorder, Bibliometrics, Fragile X syndrome, Neurodevelopmental disorders

## Abstract

Neurodevelopmental disorders (NDD), including intellectual disability, autism spectrum disorder, attention-deficit hyperactivity disorder, and specific learning disorder, are closely linked to fragile X syndrome (FXS). This study employed a bibliometric analysis to explore trends and shifts in research focusing on FXS and NDD. We extracted publication data related to FXS and NDD from the Scopus database and conducted a bibliometric analysis. This involved assessing cumulative publication trends over time, most prolific authors, geographic distribution, and co-occurrence of author keywords to identify the dominant research themes. The number of publications on FXS and NDD has steadily increased over the past decades. The United States emerged as the leading contributor, while China is becoming an important player in the field. An analysis of author keywords revealed a significant shift in research focus from molecular mechanisms to translational and clinical research. This analysis provides critical insights into the evolving research landscapes of FXS and NDD. These findings can inform future research directions, facilitate collaboration, and optimize resource allocation. Additionally, they underscore the necessity for ongoing research to deepen our understanding of these disorders and enhance treatment options for affected individuals.

## Introduction

Fragile X syndrome (FXS) is the most common inherited cause of intellectual disability (ID) and is a leading monogenic cause of autism spectrum disorder (ASD) [1]. Overall, FXS is the second most common cause of ID, after Down syndrome [2,3]. The global prevalence is predicted to be 1:4,000 to 1:7,000 in men and 1:6,000 to 1:11,000 in women [4]. The term “fragile X” refers to a cytogenetic fragile site on the long arm of the X chromosome, first described by Lubs in 1969 [5]. The genetic locus of fragile X is the 5′-untranslated region (UTR) of the *fragile X messenger ribonucleoprotein 1* gene (*FMR1*), located on Xq27.3. The association between Xq27.3 and X-linked ID was confirmed in 1991 [6]. Mutations in *FMR1* cause FXS. The 5′-UTR of *FMR1* contains unstable polymorphic trinucleotide CGG repeats, the expansion of which leads to hypermethylation and transcriptional silencing, resulting in loss of fragile X messenger ribonucleoprotein (FMRP) [7]. Apart from this, several other types of *FMR1* changes (i.e., gene deletions, intragenic deletions or duplications, and single-nucleotide variations) are responsible for the remaining (1%) molecular diagnoses of FXS [8,9].

Many behavioral symptoms identified in individuals with FXS, such as decreased social communication, social anxiety, social gaze avoidance, and stereotypical behaviors, are also observed in individuals with other causes of ASD. Furthermore, a high proportion of individuals with either FXS or ASD fit the criteria for attention-deficit hyperactivity disorder (ADHD). Males with FXS (60%) fulfill ASD criteria more commonly than females (20%), just as ASD is more common in males than females [10]. FMRP controls the translation of approximately 30% of the genes related to ASD. The overlap between FXS and ASD has a molecular basis [11].

Neurodevelopmental disorders (NDD), including ID, ASD, ADHD, and specific learning disorder, are closely associated with FXS [12]. In recent years, the study of FXS and NDD has received considerable attention, with numerous research articles published in this area, including bibliometric studies. Bibliometric analysis, a quantitative technique used to evaluate research output, can provide valuable insights into research trends, patterns, and impacts. To date, bibliometric studies have largely focused on NDD [13-17] or ASD [18-27] independently, with limited attention paid to their overlap with FXS.

A recent study by Kaplan et al. [28] examined the relationship between FXS and autism using the Web of Science database. However, a comprehensive bibliometric analysis integrating FXS with broader NDD remains limited. To address this gap in the present study, we conducted a bibliometric review of FXS and NDD using the Scopus database to analyze publication trends, leading countries and institutions, and patterns of research collaboration. In addition, a keyword analysis of publications from the past decade was performed to identify major research themes and emerging directions. These findings provide valuable insights for researchers, policymakers, and clinicians by clarifying the current research landscape and highlighting priorities for future investigations.

## Methods

### 1. Study design and tools

A comprehensive literature search was conducted using the Scopus database, one of the largest curated sources of peer-reviewed scientific literature [29]. The search strategy and screening process were inspired by PRISMA (Preferred Reporting Items for Systematic reviews and Meta-Analyses) principles to enhance transparency, reproducibility, and methodological rigor in the identification and selection of relevant publications, as illustrated in Fig. 1 [30].

To ensure high specificity and relevance, the search was restricted to the title field. This approach minimized the inclusion of studies in which FXS or NDD were not the central topics. However, publications that mentioned these terms only in the abstract or keywords may have been excluded. Accordingly, the results should be interpreted as representing the core literature directly focused on FXS and NDD rather than the entirety of potentially related research.

The search was conducted on April 8, 2026. The retrieved records were screened to exclude publications that did not meet the inclusion criteria, such as conference papers, book chapters, short surveys, notes, editorials, books, and non-English-language articles.

### 2. Data collection and analysis

In this review, we analyzed the previously published literature on FXS and NDD; therefore, ethical approval was not required. Bibliographic data retrieved from the literature search were exported in comma-separated values format for subsequent analysis. Bibliometric analyses were performed using the freely available software VOSviewer version 1.6.17 (Centre for Science and Technology Studies, Leiden University, Leiden, the Netherlands) [31], a widely used tool for constructing, analyzing, and visualizing bibliometric networks [32].

Network analyses were performed using the full-counting method with association-strength normalization, which is commonly recommended for mapping relationships among authors, countries, and keywords. Before the analysis, all records were carefully reviewed and standardized to ensure data consistency. Variations in author names and keywords were harmonized by applying a thesaurus file to merge synonymous terms, abbreviations, spelling variants, and historical terminology referring to the same concepts—e.g., ASD and “autism spectrum disorder(s),” FXS and “Fragile X syndrome,” *FMR1* and “*FMR-1*,” and mental retardation and “intellectual disability.”

Threshold values for network construction were predefined according to the objectives of each analysis and are described in the Results section. Data cleaning and standardization procedures were performed to improve the accuracy, reliability, and interpretability of the resulting bibliometric networks.

## Results

The initial search results from the Scopus database generated 1,130 documents published between 1979 and April 8, 2026 (Fig. 1). After excluding papers in languages other than English and document types other than original and review articles, 932 documents remained.

### 1. Annual publication trends

Fig. 2 illustrates the yearly distribution of publications and cumulative total publications in FXS and NDD research from 1979 to 2026. The annual number of publications remained low during the early years (1979–1995), generally below 10, with minor fluctuations. From the early 2000s, a gradual upward trend was observed, followed by a marked increase after 2005.

Research productivity peaked between 2014 and 2018, with the annual output peaking at approximately 50 publications in 2017. Although fluctuations were observed following this peak, including intermittent declines and rebounds, the overall output remained relatively high compared to earlier decades. A modest decline was observed in recent years, which likely reflects an indexing lag.

For comparative purposes, the study period was divided into an early period (1979–2013) and a recent period (2014–2026), reflecting the expansion of research output observed in the mid-2010s. This stratification was used to explore temporal changes in publication patterns and thematic developments within the field.

### 2. Most prolific authors, co-authorship, and collaboration patterns

Table 1 summarizes the most prolific authors across the two publication periods. During the first period (1979–2013), Hagerman RJ from the University of California, USA was the leading contributor with 42 publications. This was followed by Tassone F (University of California, USA), with 19 publications. Other notable contributors included Khandjian EW (Centre de recherche CERVO, Canada) with 16 publications, Bardoni B (Université Côte d'Azur, France) with 15 publications, and Mandel JL (Institut de Génétique et de Biologie Moléculaire et Cellulaire, France) with 14 publications. Several other authors, including Warren ST, Hagerman PJ, Reiss AL, and Brown WT, also contributed substantially, each with 14 publications.

A shift in authorship patterns is evident in the more recent period (2014–2026). Roberts JE (University of South Carolina, USA) led with 31 publications, followed by Hagerman RJ with 27 publications, indicating sustained prominence. Abbeduto L, Thurman AJ, and Berry-Kravis EM, all from the USA, also ranked highly, with 18, 17, and 17 publications, respectively. Additional contributors, such as Klusek J (15 publications), Sterling A (14 publications), Hogan AL (13 publications), Tassone F (11 publications), and Losh M (10 publications), further reflect the continued dominance of USA-based researchers.

To examine collaboration among authors, a co-authorship network was mapped. Colors denote a group of authors who are closely related through co-authorship links. Fig. 3A shows the co-authorship links between authors who published at least five documents between 1979 and 2013 (51 out of 1,961 authors met the threshold, resulting in 12 different node colors), and Fig. 3B shows the co-authorship links among authors who published at least five documents between 2014 and 2026 (24 out of 2,007 authors met the threshold, resulting in 10 different node colors).

As shown in Fig. 3A, several key authors formed distinct yet interconnected clusters within the co-authorship network. Khandjian EW (Canada), Bardoni B (France), and Mandel JL (France) were prominently grouped within Cluster 1 (red), reflecting a strong collaborative core. Cluster 3 (blue) is led by Hagerman RJ (USA), who is closely associated with Tassone F, Hagerman PJ, and Hessl D (all from the USA), indicating an influential and well-connected research group. Warren ST (USA), positioned in Cluster 2 (green), appears relatively distinct, suggesting a more specialized research focus. Additional notable contributors include Brown WT (USA) in Cluster 4 (yellow) and Reiss AL (USA) in Cluster 6 (cyan), both embedded within moderately connected networks. Greenough WT and Broadie K (USA) were also represented within smaller clusters, contributing to a broader collaboration landscape. The network highlights the central role of prominent USA-based researchers, along with key international contributors, particularly from Canada and France.

In Fig. 3B, the co-authorship network reveals several distinct but interconnected clusters dominated by key contributors. Cluster 1 highlights Roberts JE as a central figure closely linked with Hogan AL and other collaborators, indicating an active and cohesive research group. Cluster 2 comprises prominent authors such as Hagerman RJ, Tassone F, and Hessl D, reflecting a well-established and collaborative core within the field. Cluster 3 includes Berry-Kravis EM, Klusek J, and Losh M, suggesting another important research grouping with moderate interconnectivity. Cluster 4, featuring Abbeduto L and Thurman AJ, represents a highly collaborative subgroup with strong links. Smaller or more peripheral clusters, such as those involving Broadie K, Oliver C, and Raspa M, exhibit limited connections, indicating more specialized or less integrated research activities. The network demonstrates a shift toward multiple collaborative hubs, with sustained contributions from established authors, alongside the emergence of newer collaborative groups.

### 3. Most productive countries and collaborative patterns

Table 2 presents the top 10 most productive countries across two distinct publication periods: 1979 to 2013 and 2014 to 2026. Between 1979 and 2013, the USA emerged as the most productive country, with a substantial publication output of 312 documents, reflecting its longstanding leadership in scholarly research. Canada and the United Kingdom (UK) followed, with 54 and 43 publications, respectively, underscoring their significant contributions to the field of developmental disability research during this period. France, Italy, and Australia also featured prominently in the top-10 lists, highlighting the global distribution of research efforts in this domain.

In contrast, the publication landscape shifted during the 2014 to 2026 period, with the USA maintaining its lead but with a reduced output of 235 documents. China surged to claim the second position, with 45 publications showcasing its rapid growth and increasing prominence in developmental disability research. France and the UK retained their positions in the top five, although there were fewer publications compared to the previous period. Canada and Italy remained active contributors but showed a decline in publication output.

Furthermore, the emergence of Switzerland, Japan, and Spain in the top-10 list during the 2014 to 2026 period highlights the evolving landscape of global research collaboration and the increasing participation of countries in developmental disability research. The marked improvement in China’s position underscores the need for expanding international collaboration within the field. Table 2 illustrates the changing dynamics of scholarly publication trends in developmental disability research over time, reflecting shifts in research focus, global collaboration patterns, and the emergence of new research powerhouses alongside established leaders in the field.

To explore collaborative networks between countries in scholarly publications, a co-authorship network map was generated. Each color on the map represents a group of countries closely linked by co-authorship. The map illustrates the co-authorship relationships among countries that have contributed to at least five documents, as shown in Fig. 4. Fig. 4A covers the period from 1979 to 2013, whereas Fig. 4B represents the recent period from 2014 to 2026.

Between 1979 and 2013, 15 of 51 countries met the criteria depicted by six distinct node colors. These clusters interconnect, with Cluster 1 (red) comprising France, Italy, Belgium, Spain, and the Netherlands; Cluster 2 (green) including the USA, China, and India; Cluster 3 (blue) encompassing Australia, Canada, and Japan; Cluster 4 (yellow) involving Germany and Switzerland; and Cluster 5 (purple) comprising Sweden and the UK.

From 2014 to 2026, 19 of 61 countries fulfilled the criteria, resulting in four different node colors. All four clusters were interconnected, with the USA (Cluster 4, yellow) positioned at the center. Notably, a close collaboration is observed, particularly between Clusters 1 (red) and 2 (green), which include countries such as Italy, Germany, Japan, Belgium, Singapore, Austria, Sweden, France, Canada, Switzerland, the Netherlands, and Denmark. Additionally, Asian countries such as China and South Korea are grouped with the USA in Cluster 4, indicating their collaboration dynamics within the research landscape.

### 4. Co-occurrence of keyword analysis

Fig. 5 presents author keyword co-occurrence maps in the FXS and NDD literature, including keywords that appeared at least three times. Fig. 5A covers the period 1979 to 2013, comprising 61 of 714 keywords, whereas Fig. 5B represents 2014 to 2026, comprising 64 of 952 keywords. Table 3 summarizes the thematic evolution across clusters and the corresponding average publication years (APY) of FXS and NDD research. The APY values demonstrate a clear chronological progression in the intellectual structure of the field, reflecting a shift in research focus over time.

From 1979 to 2013, the most influential keywords were concentrated around APY 2004 to 2009, reflecting intensive efforts to identify the molecular basis of FXS, characterize FMRP function, investigate synaptic plasticity, and establish *FMR1* mutations as the central pathogenic mechanism. Clinical research during this phase largely focused on ID, screening, and diagnostic classification.

In contrast, the 2014 to 2026 period showed APY concentration between 2017 and 2023, indicating the emergence of new translational themes. These include NDD frameworks, behavioral phenotyping, language and social communication deficits, psychiatric comorbidities, genetic counseling, and pathway-oriented therapeutic targets. The appearance of recent keywords such as “genetic testing” (APY, 2023.0), “social attention” (APY, 2021.7), “NDD” (APY, 2021.5), “pragmatic language” (APY, 2020.0), “depression” (APY, 2020.3), “social anxiety” (APY, 2020.4), and “eye tracking” (APY, 2020.8) suggests that the field has increasingly shifted toward precision medicine, early detection, and clinically meaningful outcome measures.

## Discussion

The cumulative number of publications showed a steady and accelerating increase throughout the study period, with the steepest growth occurring after 2005, reflecting intensified research activity. By 2026, the cumulative total approached 950 publications, indicating sustained expansion of the field despite short-term variability in the annual output. This pattern reflects the maturation and increasing visibility of research related to FXS and NDD. Similar growth trends have also been reported in bibliometric studies of broader NDD and autism research [13-28].

Importantly, bibliometric analyses are designed to describe patterns of scientific production, collaboration, and thematic development rather than to establish the underlying causes of these trends. Therefore, although technological advances, evolving clinical priorities, and increased research investment may have contributed to the observed growth [9,33-36], the present study did not directly evaluate these factors. Similarly, the observed shifts in authorship patterns and country-level contributions indicated changes in the structure of the research community over time. The emergence of new leading authors and the increasing contribution of countries such as China suggest a more geographically diverse research landscape. However, the specific drivers of these changes could not be determined using bibliometric indicators alone. Additional analyses examining funding acknowledgments, institutional and consortium networks, and the citation impact of major research initiatives are required to better understand the mechanisms underlying these developments [37-39].

Co-occurrence keyword analysis revealed a clear evolution in the intellectual structure of FXS and NDD research, demonstrating a transition from foundational genetic discovery and molecular neurobiology to translational, clinical, and interdisciplinary research themes. This progression reflects the maturation of the field from elucidating disease mechanisms to addressing diagnosis, phenotypic characterization, intervention, and long-term outcomes.

During the early period (1979–2013), Cluster 1 was dominated by keywords related to molecular neurobiology, including “FMRP,” “RNA-binding protein,” “mRNA, translation,” “protein synthesis,” “synapse,” “synaptic plasticity,” and “hippocampus.” These terms indicate a strong emphasis on understanding the molecular and cellular mechanisms underlying FXS, particularly the role of FMRP in translational regulation and synaptic function [40,41]. The prominence of model organisms, especially *Drosophila*, and signaling pathways, such as the metabotropic glutamate receptor (mGluR) pathway, further highlights the focus on mechanistic investigations during this period. In contrast, the more recent period (2014–2026) shows an increasing prominence of keywords related to neurodevelopmental phenotypes and translational research, including “ASD,” “Down syndrome,” “Rett syndrome,” “behavioral phenotypes,” “language,” “development,” and early life outcomes. This shift suggests growing efforts to connect molecular mechanisms with clinically meaningful phenotypes and therapeutic applications [34,36,42].

A similar transition is evident in Cluster 2. Earlier studies were characterized by keywords associated with clinical descriptions and cognitive profiling, such as “intellectual disability,” “behavioral phenotypes,” “language,” “working memory,” and “cognition” [43,44]. These themes reflect the efforts to define the clinical manifestations and functional impairments associated with FXS and related conditions. However, in the later period, the cluster expanded toward systems-level neuroscience and cross-disorder biological mechanisms, as indicated by keywords such as “GABA,” “glutamate,” “mGluR,” “ERK,” “mTOR,” “synaptic plasticity,” and “neurodevelopmental disorders.” The emergence of a broader NDD terminology suggests a shift from disorder-specific investigations to a more integrated understanding of shared biological pathways across neurodevelopmental conditions.

Cluster 3 highlights the evolution of FXS genetic research. Between 1979 and 2013, the cluster was centered on “*FMR1*,” “CGG repeat,” “mutation,” and “premutation,” reflecting foundational efforts to characterize the genetic basis of FXS. During the recent period, the thematic focus has broadened to include “genetic testing,” “genetic counseling,” “metabolomics,” “prevalence,” and fragile X-associated conditions such as “primary ovarian insufficiency” and “infertility.” These developments reflect the increasing translation of genetic discoveries into clinical practice and population-level applications, while also highlighting the expanding recognition of the broader phenotypic spectrum associated with *FMR1*-related disorders [44].

Cluster 4 demonstrates a shift from diagnostic and epidemiological research to a more refined behavioral and neuropsychological characterization. Earlier keywords, including “screening,” “prenatal diagnosis,” “epidemiology,” “prevalence,” and historical diagnostic terminology, reflect efforts to identify affected individuals and establish the disease burden. In contrast, recent keywords such as “anxiety,” “depression,” “ADHD,” “social attention,” “social communication,” “executive function,” and “eye tracking” indicate a growing interest in psychiatric comorbidities, social-cognitive functioning, and objective assessment methods. These trends align with the increasing recognition of phenotypic heterogeneity [23] and the need for more sensitive outcome measures in both research and clinical settings.

Overall, the thematic evolution observed across all keyword clusters suggests that FXS and NDD research has progressed from foundational studies on gene discovery, diagnosis, and basic neurobiology to a more integrated and translational research landscape. The increasing prominence of behavioral phenotyping, genetic testing, neurodevelopmental frameworks, and pathway-oriented research reflects the field’s growing emphasis on precision medicine and individualized approaches to care. While bibliometric analyses cannot determine the factors responsible for these changes, the observed trends are consistent with broader developments reported in the literature, including advances in molecular genetics, translational neuroscience, and multidisciplinary approaches to NDD [34-36].

These findings also highlight several areas that are likely to remain important for future research, including early detection, biomarker discovery, characterization of phenotypic variability, and translation of mechanistic insights into effective interventions [35,45]. The increasing convergence of genetic, neurobiological, behavioral, and public health perspectives suggests that future progress in FXS and NDD research will depend on continued interdisciplinary collaboration and the integration of findings across multiple levels of investigation [46].

This study has several limitations that should be considered when interpreting the findings. First, the analysis was based solely on the Scopus database and English-language publications, which may have excluded relevant studies indexed in other databases or published in other languages. Second, the search strategy was restricted to article titles to maximize relevance, potentially omitting publications in which FXS or NDD were primarily discussed in the abstracts or formed the basis of the keywords. Third, as with all bibliometric studies, the findings are descriptive in nature and primarily characterize the patterns of scientific production, collaboration, and thematic development. Consequently, this analysis could not determine the causal factors underlying the observed changes in publication output, authorship patterns, research collaboration, or thematic evolution.

Future studies could strengthen our understanding of the drivers of research development by incorporating funding acknowledgment analyses, institutional- and consortium-level mapping, citation network analyses, and science policy evaluations. Such approaches would provide deeper insights into the mechanisms that shape research growth, international collaboration, and knowledge development in FXS and NDD research.

## Conclusion

Our findings highlighted a consistent increase in the number of research publications on FXS and NDD over the past few decades, indicating a growing interest in understanding these conditions. The USA emerged as a prominent contributor, with a notable shift in the most productive authors and countries over time. New players, such as China, have entered the field, reflecting changing global research dynamics. The analysis of authorship patterns also indicated changes in the most productive and influential researchers across different periods, reflecting a dynamic and evolving research community. In addition, keyword and thematic analyses revealed a thematic evolution in the research focus from defining and identifying FXS using broad diagnostic and epidemiological approaches to deeply characterizing its cognitive, behavioral, and psychiatric complexity using advanced tools and refined constructs. This evolution reflects a broader shift toward precision neurodevelopmental research, emphasizing individualized phenotyping, biomarker discovery, and targeted therapeutic strategies.

## Figures and Tables

**Fig. 1.**
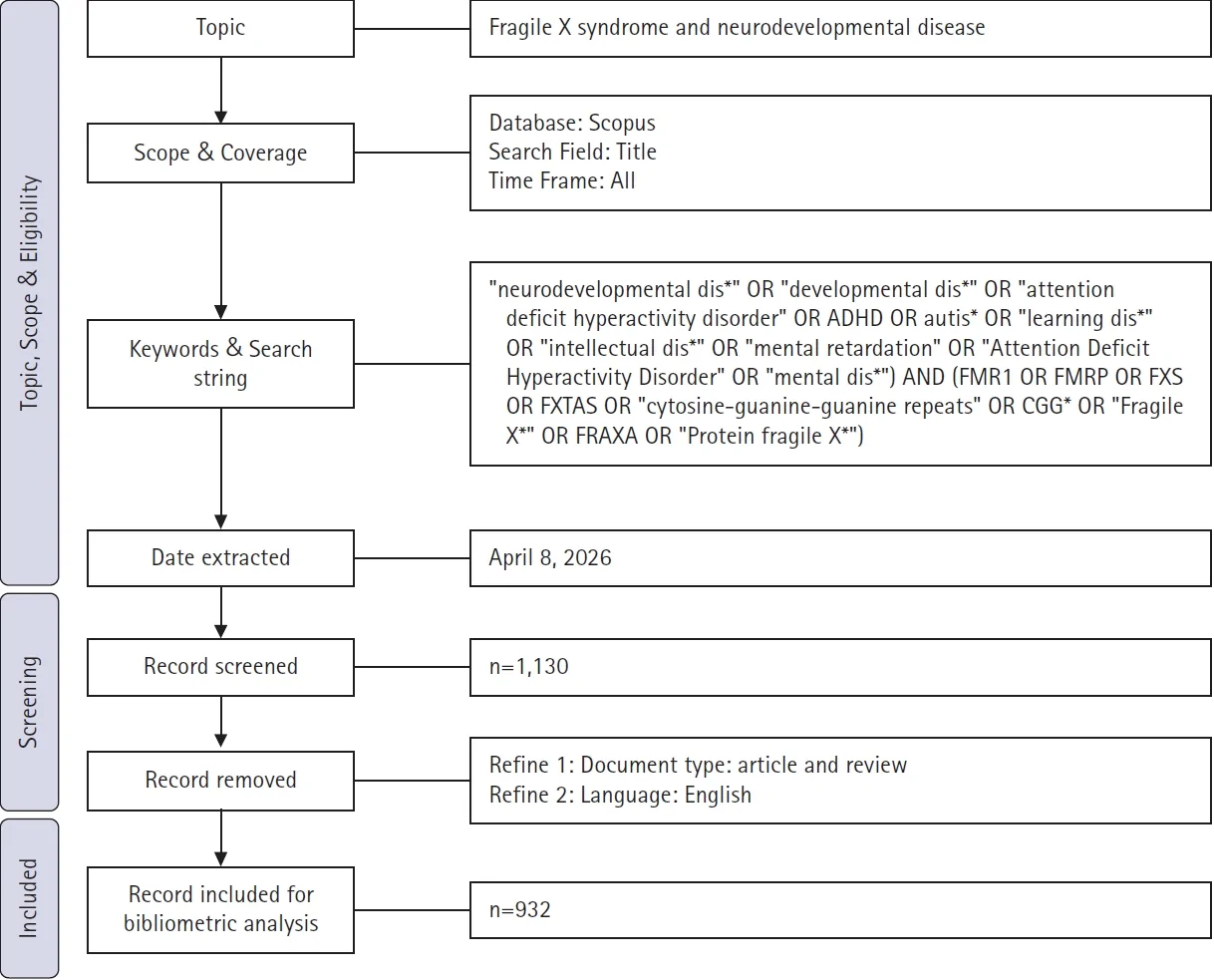
PRISMA (Preferred Reporting Items for Systematic reviews and Meta-Analyses)-inspired search and screening flow diagram [30].

**Fig. 2.**
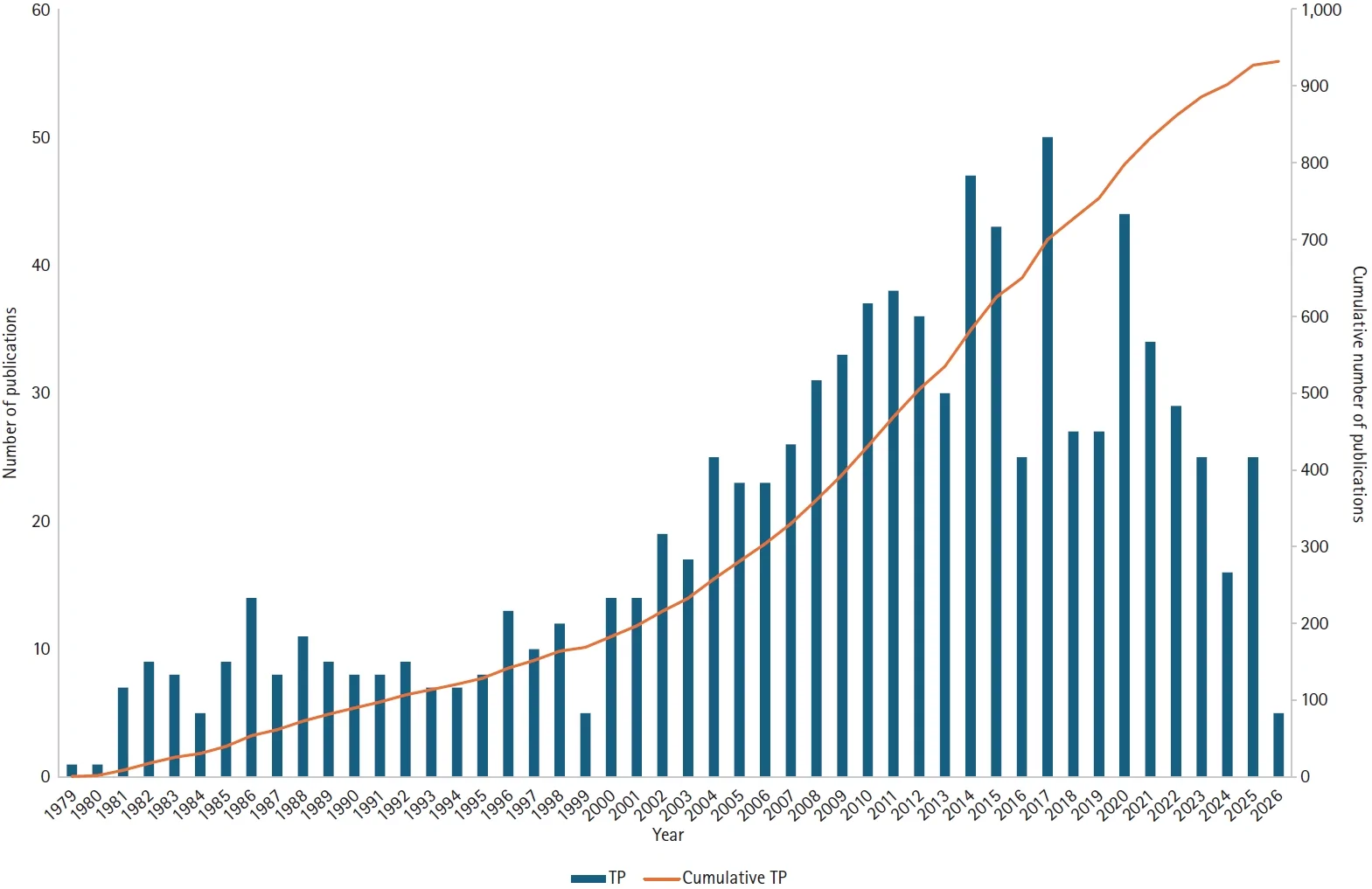
Temporal evolution of scientific publications from 1979 to 2026. TP, total number of publications.

**Fig. 3.**
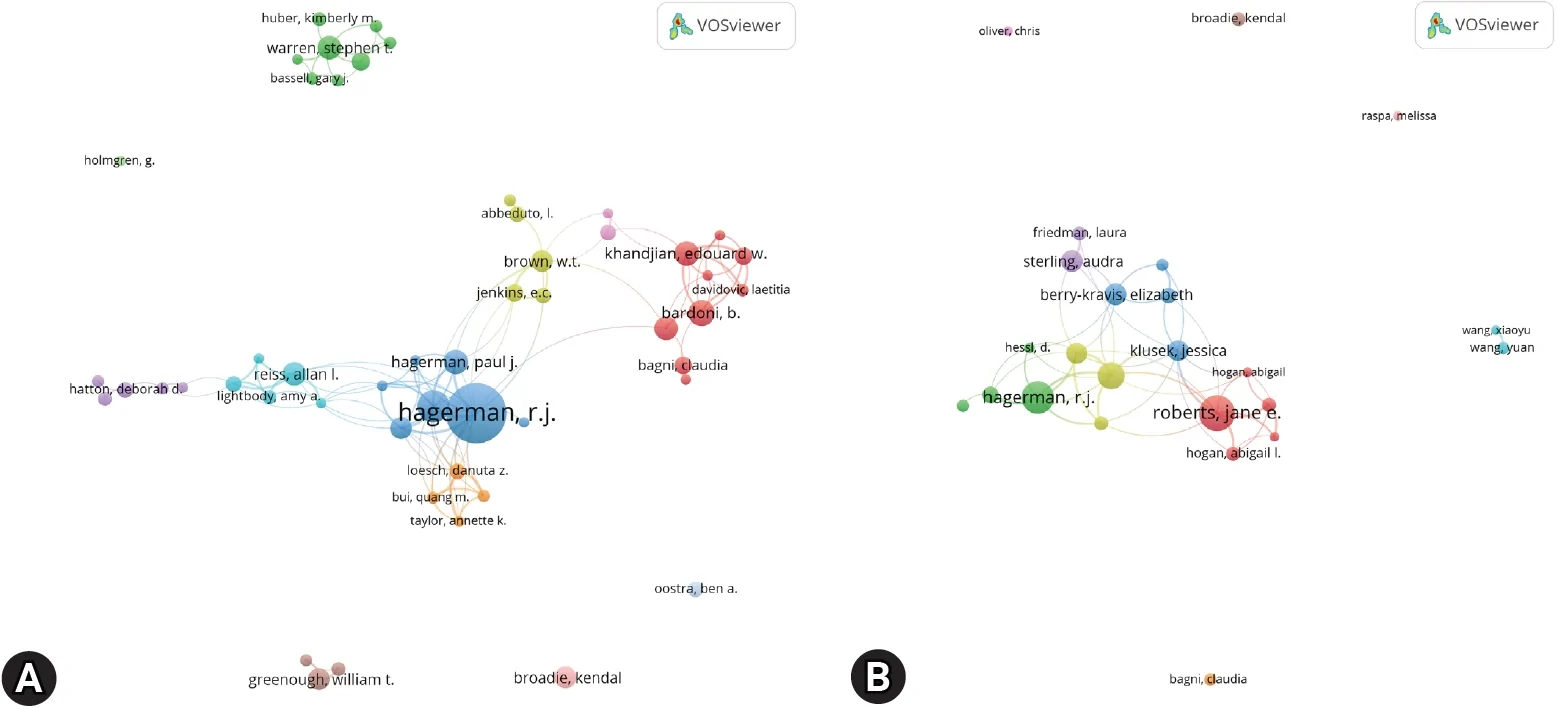
Author co-authorship networks in fragile X syndrome and neurodevelopmental disorder research: (A) 1979 to 2013; (B) 2014 to 2026.

**Fig. 4.**
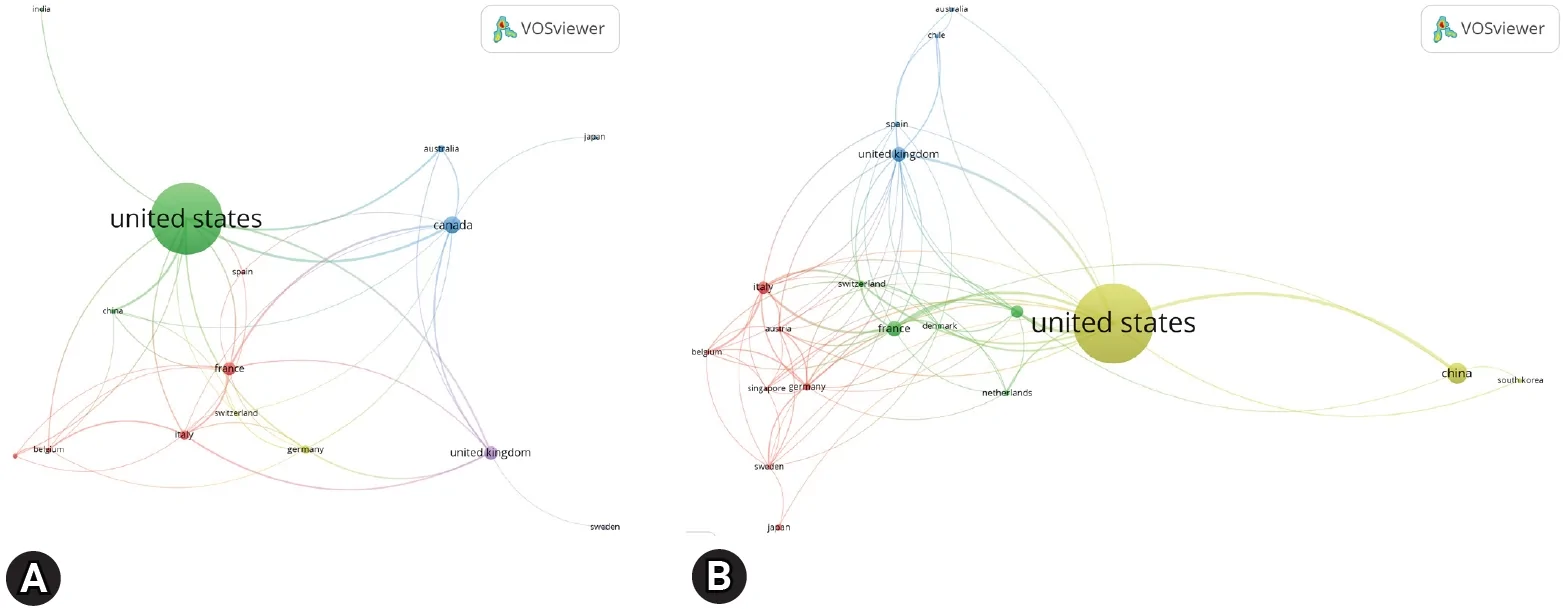
Country co-authorship networks in fragile X syndrome and neurodevelopmental disorder research: (A) 1979 to 2013; (B) 2014 to 2026.

**Fig. 5.**
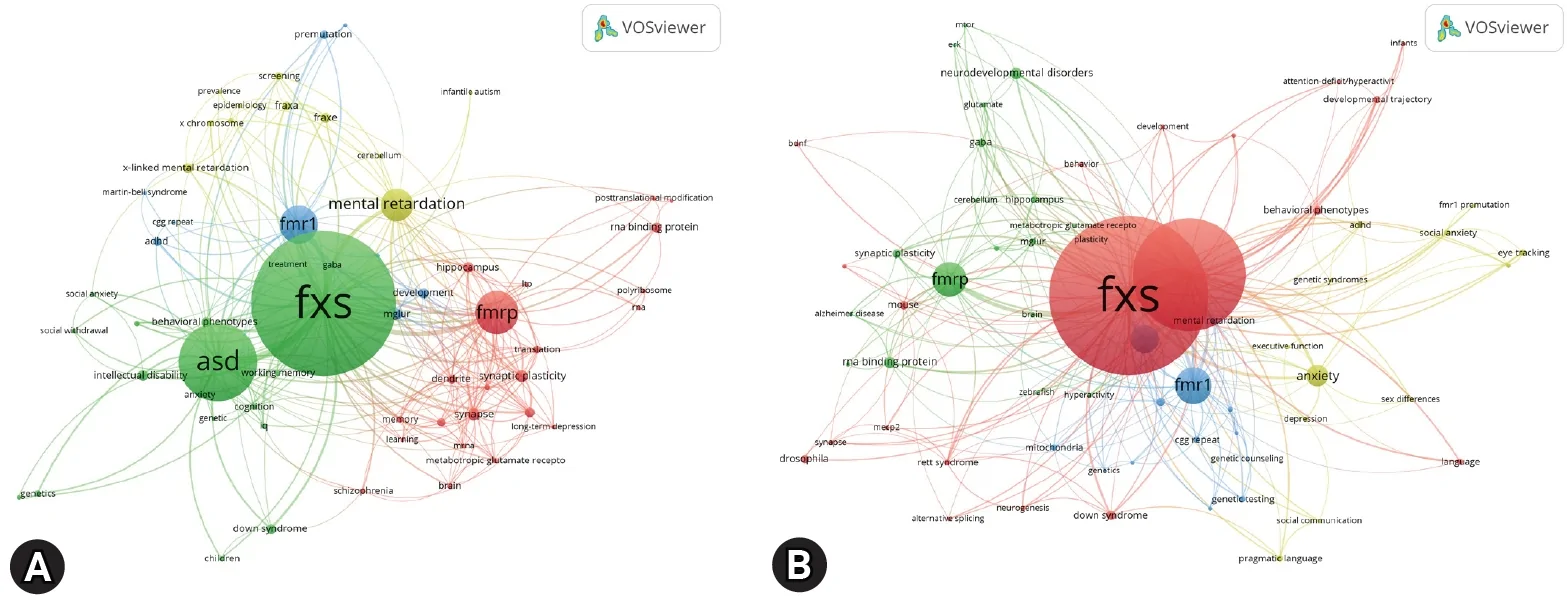
Author keyword co-occurrence networks in fragile X syndrome and neurodevelopmental disorder research: (A) 1979 to 2013; (B) 2014 to 2026.

**Table 1.** The topmost prolific authors during the two publication periods

Rank	1979–2013		2014–2026	
	Author	TP	Author	TP
1	Hagerman RJ (USA)	42	Roberts JE (USA)	31
2	Tassone F (USA)	19	Hagerman RJ (USA)	27
3	Khandjian EW (Canada)	16	Abbeduto L (USA)	18
4	Bardoni B (France)	15	Thurman AJ (USA)	17
5	Mandel JL (France)	14	Berry-Kravis EM (USA)	17
6	Warren ST (USA)	14	Klusek J (USA)	15
7	Hagerman PJ (USA)	14	Sterling A (USA)	14
8	Reiss AL (USA)	14	Hogan AL (USA)	13
9	Brown WT (USA)	14	Tassone F (USA)	11
10	Greenough WT, Broadie K, Hessl D (USA)	12	Losh M (USA)	10

TP, total number of publications.

**Table 2.** The topmost productive countries during the two publication periods

Rank	1979–2013		2014–2026	
	Country	TP	Country	TP
1	USA	312	USA	235
2	Canada	54	China	45
3	UK	43	France	32
4	France	39	UK	30
5	Italy	28	Canada	25
6	Germany	22	Italy	24
7	Australia	21	Germany	14
8	Belgium	13	Switzerland	11
9	Netherlands	12	Japan	11
10	Sweden/China	12	Spain	10

TP, total number of publications.

**Table 3.** Keyword theme evolution across clusters and APYs (1979–2013 vs. 2014–2026)

Cluster: Theme	1979–2013	2014–2026	Thematic evolution
	Keyword (APY)	Keyword (APY)	
Cluster 1: Synaptic function and molecular neurobiology	FMRP (2007.5), synapse (2008.0), synaptic plasticity (2007.1), hippocampus (2004.3), protein synthesis (2006.8), RNA-binding protein (2004.9), translation (2007.3), long-term depression (2008.3)	FXS (2018.9), ASD (2019.2), Down syndrome (2019.9), behavioral phenotypes (2018.1), mouse (2018.3), development (2018.7), language (2019.0), Rett syndrome (2019.0)	Early studies focused on the molecular role of FMRP and mechanisms of synaptic dysfunction. After 2014, the cluster expanded toward translational neuroscience, linking synaptic abnormalities with behavioral phenotypes, NDDs, and developmental outcomes.
Cluster 2: Genetics, NDD, and clinical phenotypes	FXS (2005.3), ASD (2006.3), intellectual disability (2010.1), behavioral phenotypes (2004.5), Down syndrome (2008.9), cognition (2009.0), working memory (2010.5), language (2011.0)	FMRP (2018.2), NDDs (2021.5), RNA binding (2019.7), GABA (2016.3), synaptic proteins (2017.0), hippocampus (2015.2), Alzheimer disease (2017.3), brain (2017.3)	The focus shifted from describing cognitive and developmental manifestations of FXS toward understanding shared biological mechanisms across neurodevelopmental and neurodegenerative disorders. The appearance of Alzheimer disease and broader NDD terminology suggest increased interdisciplinary integration.
Cluster 3: *FMR1* genetics and experimental models	*FMR1* (2006.6), mGluR (2009.0), development (2004.9), ADHD (2006.3), premutation (2007.7), CGG repeat (2008.3), GABA (2009.7)	*FMR1* (2019.6), intellectual disability (2019.8), premutation (2016.7), CGG repeat (2016.6), genetic testing (2023.0), mitochondria (2017.8), genetic counseling (2020.7)	Research evolved from gene discovery and mechanistic hypotheses (e.g., mGluR theory) toward clinical genetics, carrier identification, genetic counseling, and precision medicine. The emergence of genetic testing as one of the newest keywords (APY 2023) highlights increasing clinical translation.
Cluster 4: Epidemiology, diagnosis, and behavioral outcomes	Mental retardation (2002.7), FRAXE (1999.5), FRAXA (1999.6), screening (2003.2), prenatal diagnosis (1989.8), epidemiology (1991.0), infantile autism (1987.3), prevalence (1997.3)	Anxiety (2019.1), ADHD (2019.6), eye tracking (2020.8), pragmatic language (2020.0), social anxiety (2020.4), depression (2020.3), executive function (2015.3), social attention (2021.7)	Earlier studies concentrated on diagnosis, epidemiology, and syndrome identification. Recent work increasingly emphasizes behavioral assessment, social cognition, psychiatric comorbidities, and digital/experimental assessment methods such as eye tracking.

APY, average publication years; FMRP, fragile X messenger ribonucleoprotein; FXS, fragile X syndrome; ASD, autism spectrum disorder; GABA, gamma-aminobutyric acid; NDD, neurodevelopmental disorders; mGluR, metabotropic glutamate receptor; *FMR1*, *fragile X messenger ribonucleoprotein 1* gene; CGG, cytosine-guanine-guanine; ADHD, attention-deficit hyperactivity disorder; FRAXE, fragile X site E at Xq28 (CGG repeat expansion in the *AFF2* gene); FRAXA, fragile X site A at Xq27.3 (CGG trinucleotide repeat expansion in the *FMR1* gene).
